# Routine Use of the “Penumbra” Thrombectomy Device in Myocardial Infarction: A Real-World Experience—ROPUST Study

**DOI:** 10.1155/2022/5692964

**Published:** 2022-03-26

**Authors:** Nour Tashtish, Tarek Chami, Tony Dong, Besher Chami, Sadeer Al-Kindi, Haytham Mously, Scott Janus, Tarek Hammad, Mehdi H. Shishehbor, Anjan Gupta

**Affiliations:** ^1^Harrington Heart and Vascular Institute, University Hospitals Cleveland Medical Center, Cleveland, OH, USA; ^2^Department of Medicine, University Hospitals Cleveland Medical Center, Cleveland, OH, USA; ^3^Department of Medicine, Aleppo University Hospital, Aleppo, Syria

## Abstract

**Objectives:**

Evaluation of the safety and efficacy of the Penumbra device as an adjunct to percutaneous coronary intervention (PCI) in patients with myocardial infarction (MI) and a large thrombus burden that requires thrombectomy.

**Background:**

For patients with acute MI, PCI is the primary reperfusion method. Large thrombus burden has always been a limitation of successful reperfusion. However, the use of current aspiration devices has been associated with an increased incidence of stroke.

**Methods:**

We performed a retrospective chart review at the University Hospitals Medical Center in Cleveland. Our study included data from patients who underwent PCI for ST segment elevation myocardial infarction (STEMI) and non-ST segment elevation myocardial infarction (NSTEMI) assisted by the Penumbra Cat RX device (a wide-lumen thrombus aspiration catheter) between May 2019 and February 2021. The primary outcome was the final thrombolysis in myocardial infarction (TIMI) flow. The secondary endpoints were a composite of adverse cardiac events at 6 months. About 50% of the patients did undergo transfemoral PCI as per preference of individual operators. The Penumbra thrombectomy device can be used both by radial and femoral approach and does not need any different guide catheter use.

**Results:**

TIMI flow 3 was achieved in 111 patients (90.2%). The secondary endpoint occurred in 11 patients (8.9%, 3 MI, 8 heart failure hospitalizations). There were no stroke events or device-related complications. The door-to-balloon time was not affected by usage of the Penumbra device. Failure in the restoration of TIMI 3 flow was associated with the use of balloon angioplasty prior to the application of the Penumbra device, leading to distal embolization.

**Conclusions:**

The Penumbra Cat RX provides safe and effective thrombus removal with better clinical outcomes, even in high-risk patients with acute coronary syndrome.

## 1. Introduction

Percutaneous coronary intervention (PCI) is the primary reperfusion method for patients with acute myocardial infarction (MI). Successful reperfusion, however, may be limited by thrombus burden and/or microvascular dysfunction [1]. Thrombectomy devices could mitigate some of these limitations through thrombus extraction and preventing distal embolization, but data supporting their safety and efficacy are controversial. The use of current aspiration devices available in the market (prior to the approval of Penumbra) has been associated with an increased incidence of stroke [2].

Accordingly, the 2015 American College of Cardiology/American Heart Association guidelines assigned aspiration thrombectomy as a class III recommendation for routine upfront use before PCI and a class IIb recommendation in selected cases or as a bailout strategy [3]. The Indigo® System CAT RX device (Penumbra Inc, Alameda, CA, USA) is a new mechanical device with an atraumatic, wide-lumen catheter that delivers continuous vacuum suctioning. In this study, we evaluated the safety and efficacy of the Penumbra device as an adjunct to PCI in patients with MI who had large thrombus burden that required thrombectomy across our health system.

## 2. Materials and Methods

We performed a retrospective chart review at the University Hospitals Cleveland Medical Center using the data of all patients who underwent interventions for ST-Elevation Myocardial Infarction (STEMI) and non-ST-Elevation Myocardial Infarction (NSTEMI) between May 2019 and February 2021. The study protocol was approved by our institutional review board (STUDY20211345).

The primary outcome was the final thrombolysis in myocardial infarction (TIMI) flow. The secondary endpoints were a composite of adverse cardiac events at six months, which was defined as a composite of death, recurrent myocardial infarction, and heart failure hospitalization. Safety outcomes included stroke and device-related complications.

A high thrombus burden was defined as TIMI thrombus grade four or five by the physician's visual estimate after the guidewire crossed the target lesion.

### 2.1. Patients

All patients presenting with acute coronary syndrome (STEMI and NSTEMI) were included in the chart review. The Penumbra thrombectomy device was used at the discretion of the individual operators in patients who had visible thrombus in the coronary arteries at the time of initial procedure. The majority of patients received 4000 units of heparin and were loaded with 180 mg of ticagrelor and 325 mg of aspirin prior to the procedure, as per our system protocol. The use of IIb/IIIa agents was performed at the discretion of the operator.

### 2.2. Thrombectomy Procedure

**Figure 1:**
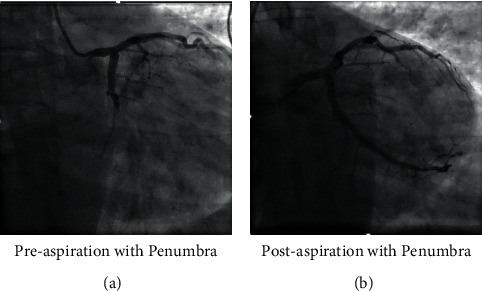
Case 1: 42-year-old male patient presenting with inferolateral STEMI. STEMI, ST segment elevation myocardial infarction.

**Figure 2:**
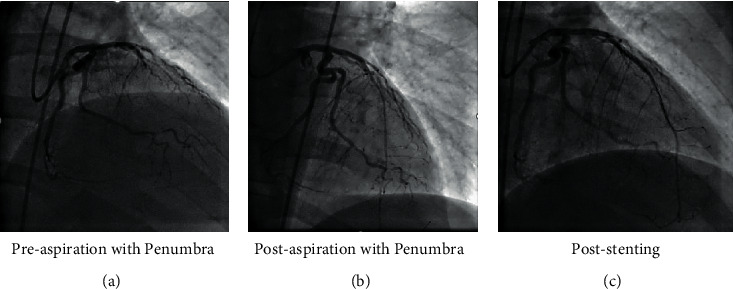
Case 2: 55-year-old male presenting late after anterior STEMI. STEMI, ST segment elevation myocardial infarction.

Thrombectomy with the Penumbra device was performed according to the manufacturer's instructions. A 6 F Cat RX catheter was used in all patients. After the lesion was crossed with a 0.014-inch guidewire, the Penumbra catheter was advanced with the guide catheter (6French), and aspiration was initiated proximal to the lesion. The guide catheter was kept closely engaged with the coronary ostia to prevent systemic embolization. The device was kept in a suction mode until it was outside the body. The guide catheter was thoroughly aspirated and flushed, and back bleeding was ensured before contrast injection. The number of passes was left to the operator's discretion (Figure 1 and 2).

The first two investigators (TC and TD) adjudicated the outcomes by a retrospective chart review.

### 2.3. Statistical Methods

Patient demographics and follow-up data were collected during the initial consultation and by reviewing the medical records. Statistical analyses were performed using IBM SPSS Statistics for Windows, version 23 (IBM Corp, Armonk, NY, USA).

A number of covariates were included in the study, including age, sex, diabetes, hypertension, hypercholesterolemia, previous PCI, previous MI, preprocedure TIMI flow, postprocedure TIMI flow, glycoprotein IIb/IIIa use, presence of cardiogenic shock, and method of vascular access.

## 3. Results

**Figure 3:**
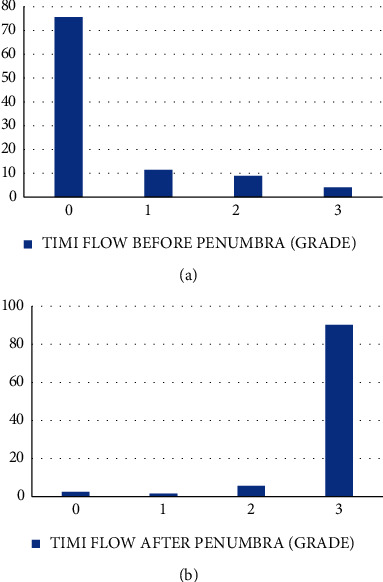
(A) TIMI flow before Penumbra (grade). (B) TIMI flow after Penumbra (grade). TIMI, thrombolysis in myocardial infarction.

Between May 2019 and February 2021, 3750 patients presented as acute coronary syndrome (ACS); 956 (25.5%) were ST-elevation MI (STEMI); 2794 (74.5%) were non-ST-elevation MI (NSTEMI); 1468 (39.1%) patients underwent PCI. 123 nonconsecutive patients underwent PCI for acute MI assisted by the Penumbra device across our health system (Table 1). The use of adjunctive antithrombotic medications or other devices was left to the discretion of the treating physician. The mean age was 60 ± 12.7 years; 96 (77.5%) were males and 89 (72.4%) presented with ST-elevation MI on ECG. A TIMI flow 0 or 1 with visible thrombus was observed in 107 patients (87%) (Figure 3). The most common culprit vessel was the right coronary artery (45.5%), followed by the left anterior descending artery (31.7%). Radial access procedures were performed in 65 patients (52.8%). All patients had a high thrombus burden (grade 4 or 5) (Table 2).

At the end of the procedure, TIMI flow 3 was achieved in 111 patients (90.2%). The secondary endpoint occurred in 11 patients (8.9%, 3 MI, 8 heart failure hospitalizations) and deaths occurred 14 patients (11.3%). Eight patients had cardiogenic shock either at presentation or shortly after the PCI. There were no stroke events or device-related complications. Ten (8.1%) had died in the hospital, and four patients (3.2%) died within >30 days and were non-cardiac-related deaths. The cause of death is further described in the Supplementary Material.

Among the 12 patients (9.8%) in whom TIMI 3 was not achieved, 9 patients (70%) had received balloon dilatation before the use of the Penumbra CATRx; the remaining two patients had small vessels, and one patient had severe ectatic disease of the coronary arteries (Figure 3). The mean door-to-balloon time was 65.51 ± 64 min. The median duration was 54 (32–75) min. Among the STEMI patients who underwent follow-up electrocardiography, 60 patients (66.3%) had a resolved ST segment elevation within 24 hours (Table 2).

## 4. Discussion

In this study, we report the results of the use of the Penumbra thrombectomy device during primary PCI in patients with STEMI and extensive thrombus burden. This is the largest series of patients reported in the literature in whom the Penumbra device was used.

In this single-system series, no stroke events were noted after the use of the Penumbra device, and no device-related complications were observed in our cohort. We also performed a search in the Manufacturer and User Facility Device Experience (MAUDE) database between January 2018 and December 2020. Entries were manually screened, and no device-related complications were found.

In our study, the majority of deaths occurred in patients who presented with or developed cardiogenic shock, most commonly due to late presentation and proximal large vessel occlusion affecting a large area of the myocardium. The Penumbra catheter was successfully used to restore coronary artery flow in this high-risk population.

In previous trials, thrombectomy with a syringe-based aspiration catheter failed to show a significant reduction in cardiovascular events including mortality [4]. These devices were also associated with a higher risk of stroke compared with conventional PCI, probably due to an inconsistent vacuum force that decreases the efficacy and increases the risk of systemic embolization during catheter removal.

However, the Penumbra device is attached to a continuous vacuum suction motor. This allows for a more consistent suction power during the procedure, making it more effective in reducing the clot burden and decreasing the risk of embolic stroke. Despite these advantages, there have been limited real-world data published on the adjunctive use of the penumbra during PCI. In a series of 59 patients who underwent PCI with adjunctive penumbra use, Mathews et al. demonstrated excellent postprocedural TIMI flow along with minimal complications and no ischemic stroke events [5].

We strongly believe that the Penumbra CatRx device should be used before any balloon angioplasty in patients with high thrombus burden as the initial use of balloon angioplasty leads to distal embolization of the thrombus, resulting in no flow or poor distal flow after the procedure. In our experience, the use of a balloon may lead to failure in achieving a TIMI 3 flow postdevice in the majority of cases. In our series, in all cases where balloon was used, prethrombectomy was due to personal choice of the performing physician and not due to difficulty in crossing the lesion with the Penumbra catheter. However, in our experience, it is advisable to use a small profile balloon for predilatation in case if there is failure to deliver the thrombectomy device on initial attempt.

The device is easy to use, easy to set up, and does not affect the door-to-device time, as shown in our study where the mean door-to-device time was below the ideal national average of 90 minutes recommended by the American Heart Association (AHA) [3, 6].

Infusion of IIb/IIIa receptor antagonists was used in 30% of the patients at the discretion of the operator as an adjunct to thrombectomy procedure in case of failed or suboptimal results of aspiration thrombectomy.

Prior studies on the use of aspiration thrombectomy did not include patients with NSTEMI, as it was thought that the clot associated with NSTEMI may not be amenable to aspiration thrombectomy. Furthermore, it was shown in one study that it did not improve clinical outcomes at 12 months [7]. However, these studies used first-generation noncontinuous aspiration devices. In our study, we included patients having NSTEMI with a large thrombus burden in whom we successfully used the Penumbra CatRx device for thrombus aspiration.

### 4.1. Limitations

Our study has some limitations, including the absence of a control group and the small sample size from a single-system retrospective experience, despite the presence of nine hospitals that range from community practice to quaternary centers in our health system. Future studies with larger sample sizes are necessary to compare the outcomes of these patients with those who underwent PCI alone or those who underwent thrombectomy with other adjunctive devices or medications. Another limitation is that no data have been reported on (a) median aspiration time, (b) median access-to-reperfusion time, and (c) aspiration power (size and number of debris).

The CHEETAH trial is a multicenter, prospective trial to assess the CAT RX catheter's safety and performance in patients with acute coronary occlusion and high thrombus burden. The trial was recently presented at the Transcatheter Cardiovascular Therapeutics (TCT) meeting, and it demonstrated 96.5% freedom from major adverse cardiac events (MACE) at 30 days with 0 device-related adverse device effects (ADEs) including stroke. The device successfully removed the thrombus with 85% TIMI 2 or 3 flow in 85% of the patients and then TIMI 3 flow in 97.5% of the patients. These results are consistent with the findings of our study [8, 9].

## 5. Conclusion

In conclusion, we present the largest real-world experience of penumbra use for adjunctive thrombectomy in patients with STEMI and NSTEMI and hypothesize that it is a useful device to help in the complete evacuation of the thrombus in most cases and it is safe as evidenced by the zero-stroke rate even in a high-risk population. We need randomized control studies in future to establish its superiority to balloon angioplasty and stent alone in patients with extensive thrombus in the coronary arteries and acute coronary syndromes.

### 5.1. Perspectives

A large thrombus burden has always been a limitation of successful reperfusion in acute myocardial infarction due to distal embolization and microvascular dysfunction. Traditionally, the use of aspiration devices has been associated with an increased incidence of stroke and unsuccessful thrombectomy due to limitation of the force of passive aspiration. In our study, we are reporting that the use of Penumbra aspiration devices for acute myocardial infarction has been associated with no incidence of stroke and successful restoration of flow. Further prospective randomized trials will be needed to show its usefulness against current standards of care.

## Figures and Tables

**Table 1:** Baseline characteristics of patients.

Demographic characteristics		*N* = 123
Age (years, IQR)		60 (54, 70)

Sex	Male	95 (77.2%)
	Female	28 (22.8%)

Race	Black	19 (15.4%)
	White	95 (77.2%)
	Other	9 (7.4%)

Risk factors	Obesity (BMI>30)	63 (51.2%)
	Hypertension	101 (82.1%)
	Dyslipidemia	108 (87.8%)
	History of heart failure	11 (8.9%)
	Diabetes mellitus	45 (36.6%)
	History of smoking	101 (82.1%)
	Prior stroke	5 (4.06%)
	Prior myocardial infarction	7 (5.9%)
	Prior percutaneous coronary intervention	6 (10.5%)

Presentation	NSTEMI	33 (26.8%)
	STEMI	89 (72.4%)

Medication	Beta blockers	51 (89.5%)
	ACEi/ARB	30 (52.6%)
	Statin	56 (98.2%)
	Aspirin	56 (98.2%)
	Calcium channel blockers	10 (17.5%)

**Table 2:** Procedural details.

Periprocedural Medications	Aspirin	*N* = 123
		100 (81.3%)
	Cilostazol	2 (1.6%)
	Heparin	69 (56.1%)
	Clopidogrel	11 (8.9%)
	Ticagrelor	85 (69.1%)
	Prasugrel	6 (4.9%)
	GP IIb/IIIa	38 (30.9%)
	Cangrelor	11 (8.9%)

Procedural characteristics	Access site for PCI	
	Femoral	58 (47.2%)
	Radial	65 (52.8%)
	Intracoronary imaging	
	None	100 (81.3%)
	Intravascular ultrasound (IVUS)	9 (7.3%)
	Optical coherence tomography (OCT)	14 (11.4%)
	Contrast volume (mL, IQR)	122.5 (100, 167.5)
	Radiation time (min, IQR)	11.7 (7.5, 19.5)
	Door-to-balloon time (min, IQR)	54 (32–75)
	Q wave on presentation	54 (43.9%)
	Resolving ST elevation within 24 hours in STEMI patients	59 (66.3%)

Culprit vessel	Left anterior descending artery (LAD)	39 (31.7%)
	Right coronary artery (RCA)	56 (45.5%)
	Left circumflex artery (LCx)	11 (8.9%)
	Vein graft	11 (8.9%)
	Left main (LM)	1 (0.8%)
	Obtuse marginal artery (OM)	3 (2.4%)
	Others	3 (2.4%)

Dominance	Left dominance	8 (6.5%)
	Right dominance	108 (87.8%)
	Codominance	7 (5.7%)

TIMI flow (baseline)	0	93 (75.6%)
	1	14 (11.4%)
	2	11 (8.9%)
	3	5 (4.1%)

TIMI flow (final)	0	3 (2.4%)
	1	2 (1.6%)
	2	7 (5.7%)
	3	111 (90.2%)

Stenosis degree (before)	≤95%	18 (14.6%)
	≥99%	105 (85.3%)

Stenosis degree (after)	0%	86 (69.9%)
	>0%	37 (30%)

## Data Availability

The data used to support the findings of this study are available from the corresponding author upon request.

## Supplementary Materials

Supplementary Materials Supplementary Table 1: cause of death.
