# Supplementary material for: Routine Use of the “Penumbra” Thrombectomy Device in Myocardial Infarction: A Real-World Experience—ROPUST Study
Source: J Interv Cardiol. 2022 Mar 26;2022:5692964. doi: 10.1155/2022/5692964 (PMC8976598; doi:10.1155/2022/5692964)
Supplement: Supplementary Materials — Supplementary Table 1: cause of death. [file 5692964.f1.docx]

Supplementary Table 1. Cause of death

| Cause of death | Time since index events (days) |
| --- | --- |
| STEMI, pulseless VT in the catheterization lab | 0 |
| STEMI, cardiogenic shock | 1 |
| STEMI, cardiogenic shock | 1 |
| STEMI, cardiogenic shock | 1 |
| Intracranial hemorrhagic shock | 1 |
| Cardiogenic shock, VT arrest | 1 |
| STEMI, cardiogenic shock | 3 |
| Sepsis with possible coronary vasospasm | 3 |
| COVID, PEA arrest, and hypoxia | 7 |
| STEMI, VT arrest on presentation | 9 |
| Unknown death | 57 |
| PEA arrest, possibly DKA | 85 |
| Sepsis transitioned to hospice | 95 |
| Large Right occipital Intracranial hemorrhage | 97 |

STEMI: ST-elevation myocardial infarction

VT: Ventricular tachycardia

COVID: Coronavirus disease of 2019

PEA: Pulseless electrical activity

DKA: Diabetic ketoacidosis
